# Bodily Carriage

**Published:** 1876-01

**Authors:** 


### BODILY CARRIAGE.

"A dying man can do nothing easy," as he spilled something which was given him to swallow, were the last recorded words of him who in life had "tamed the lightning's wing," and

"bottled the thunders of Omnipotence." But it would seem an easy matter for a sane man or woman in good health *to sit down* properly. And yet not one in a multitude does it. Far-seeing mothers sometimes succeed in beating it into the heads of thoughtless daughters, by virtue of extraordinary perseverance, —as a means of getting a husband! for who ever married a stoop-shouldered or humpbacked girl? As for the sons, they are left to take their chances, and assume any shape which circumstances may determine. But it helps vastly in our efforts to accomplish laudable objects to have a clear and adequate reason to second our endeavors.

Who does not dread and hate the very name of "*Consumption?*" It does not come suddenly. It begins in remote months and years agone, by imperfect breathing; by want of frequent and full breaths, to keep the lungs in active operation. In time, the lungs swell out a quarter or one-third less than they ought to do; consequently the breast flattens, the arms bend forwards and inwards, and we have the round or high shoulder, so ominous in a doctor's eye. As consumptives *always* bend forward, and as men in high health, candidates for aldermanic honors sit and walk and stand erect,—*physically!* the erect position must be antagonistic of consumption, and consequently should be cultivated, sedulously cultivated in every manner practicable; cultivated by all, men, women and children. If we can promote this culture without interfering with the ordinary business of life, and without its costing a dollar, a valuable point is gained; and considering the importance of the subject, we shall not think ourselves to have lived in vain, if this article shall be practically adopted by any considerable number of our readers.

No place is so well adapted to secure an erect locomotion as a large city; the necessity is ever present for holding up the head; if a man does not do it, he will in any walk along a principal street knock his brains out; or if he be unusually hard-headed, knock out the brains of some less gifted pedestrian. Instead of giving all sorts of rules about turning out the toes, and straightening up the body, and holding the shoulders back, all of which are impracticable to the many, because soon forgotten, or of a feeling of awkwardness and discomfort which procures a willing omission; all that is necessary to secure the object, is to *hold up the head and move on!* letting the toes and shoulders take care of themselves. Walk with the chin but slightly above a horizontal line, or with your eye directed to things a little higher than your own head. In this way you walk properly, pleasurably, and without any feeling of restraint or awkwardness. If any one wishes to be aided in securing this habitual carriage of body, accustom yourself to carry the hands behind you, one hand grasping the opposite wrist. Englishmen are admired the world over for their full chests, and broad shoulders, and sturdy frames, and manly bearing. This position of body is a favorite with them, in the simple promenade in the garden or gallery, in attending ladies along a crowded street, in standing on the street, or in public worship.

Our young men seem to be in elysium when they can walk arm-in-arm with their divinities. Now young gentlemen, you will be hooked on soon enough without anticipating your captivity. While you are free, *walk right!* IN ALL WAYS; and when you are able, get a manly carriage, and take our word for it, it is the best way in the world to *secure* the affectionate respect of the woman you marry. Did you ever know any girl worth having, who could wed a man who mopes about with his eyes on the ground, making of his whole body the segment of a circle, bent on the wrong side. Assuredly, a woman of strong points, of striking characteristics, admires, beyond a handsome face, the whole carriage of a man. Erectness being the representative of courage and daring, it is this which makes a man of "*presence.*"

Many persons spend a large part of their waking existence in the sitting position. A single rule, well attended to, in this connection, would be of incalculable value to multitudes,—*use chairs with the old-fashioned straight backs, a little inclining backwards!* and sit with the lower portion of the body close against the back of the chair at the seat; any one who tries it, will observe in a moment a grateful support to the whole spine. And we see no reason why children should not be taught from the beginning to write, and sew, and knit, in a position requiring the lower portion of the body and the shoulders to touch the back of the chair all the time.

A very common position in sitting, especially among men, is with the shoulders against the chair back, with a space of several inches between the chair back and the lower portion of the spine, giving the body the shape of a half hoop; it is the instantaneous, instinctive, and almost universal position assumed by any consumptive on sitting down, unless counteracted by an effort of the will; hence parents should regard such a position in their children with apprehension, and should rectify it at once.

The best position after eating a regular meal is, to have the hands behind the back, the head erect, in moderate locomotion, and in the open air, if the weather is not chilly. Half an hour spent in this way after meals, at least after breakfast and dinner, would add health and length of days to women in easy life, and to all sedentary men. It is a thought which richly merits attention. As to the habit which many men have of sitting during prayer, in forms of worship not requiring it, with the elbows extended along the back of the pew, and forehead resting on the arms, we will only say in passing, that besides being physiologically unwise and hurtful, it is socially an uncourteous and indelicate position; while in a religious point of view it is an unpardonable irreverence; a position which no man with the feelings of a gentleman, unless an invalid, can possibly assume, and we wonder that it is a practice of such general prevalence. It is a position which we venture to affirm, is in almost every instance the dictate of bodily laziness or religious sleepiness or indifference. Women are not required to stand in prayer; it is physiologically hurtful; they should sit or kneel.

